# Two pathways for venom toxin entry consequent to injection of an Australian elapid snake venom

**DOI:** 10.1038/s41598-019-45022-4

**Published:** 2019-06-13

**Authors:** Dirk F. van Helden, Peter J. Dosen, Margaret A. O’Leary, Geoffrey K. Isbister

**Affiliations:** 1 School of Biomedical Sciences & Pharmacy, University of Newcastle, Callaghan, New South Wales Australia; 2 Clinical Toxicology Research Group, University of Newcastle, Callaghan, New South Wales Australia

**Keywords:** Physiology, Health care

## Abstract

Here we test and refute the hypothesis that venom toxins from an Australian elapid, the Eastern Brown snake (*Pseudonaja textilis*, PTx), solely require lymphatic transport to enter the circulation. Studies were made using anaesthetised non-recovery rats in which a marker dye (India ink) or highly potent PTx venom was injected into the hind paw. The studies required a means of inhibiting lymphatic function, as achieved by cooling of the test hind limb to low temperatures (~3 °C). Maintained entry of a non-lethal dose (0.15 mg/kg) and respiratory arrest consequent to injection of a lethal dose (1 mg/kg) of PTx venom at these low temperatures indicate that venom including toxin components enter the circulation directly via the vascular system, a process facilitated by, but not dependent on, lymphatic transport. Notably, the venom had a direct effect on vascular permeability markedly increasing this to allow extravasation of plasma albumin (MWt ~60 kDa).

## Introduction

Snakebite remains a major problem in many countries. Current estimates are that there are some 2.7 million significant envenomings per year causing an estimated 81,000 to 137,000 deaths/year and approximately 3 times more disabilities including amputations^1–11^. While the use of antivenom remains a highly successful treatment, appropriate first aid is important and may give time for victims to reach hospital for antivenom treatment. Unfortunately, effective first aid procedures are poorly implemented, with greatest success in Australia for elapid snakebite through use of the pressure bandaging with immobilisation technique (PBI)^12–14^. The technique is used for bites to limbs the most common site of snakebite, which accounts for 94% of snakebite cases^15^. Unlike the tourniquet, PBI does not cause major ischaemia and is considered safe in humans. It is based on applying a bandage at pressures of about 60 mm Hg, which is below the pressure for arterial blood flow, but above that for lymphatic flow when used together with immobilization^12,14,16^. However, even this procedure is either not applied or applied badly, with evidence that only 15% of untrained and 50% of trained helpers successfully apply PBI^17^. A related snakebite first aid procedure that may be even better, as it could also be used on torso bites, is the “Pressure Pad” technique^18,19^. However, this is rarely used and in Australia, unlike PBI, it has not been ratified by the Australian National Health & Medical Research Council.

A further approach, which could be used as an adjunct to PBI or the pressure pad methods, is use of skin permeable agents that inhibit the absorption of venom into the circulation through the lymphatics. Some success has been achieved using this approach at least for an Australian elapid venom from *Pseudonaja textilis* (PTx)^20,21^. The approach was based on the hypothesis that PTx venom toxins entered the circulation by uptake through the lymphatics and hence skin permeable agents that block lymphatic function would provide a useful first aid. The assumption was that due to the shortness of elapid fangs, venom would be injected subcutaneously, where there are many end (i.e. “initial”) lymphatics to absorb the venom from where it would pass into the larger superficial collecting lymphatics, vessels that are readily accessible by skin permeable agents that inhibit the heart-like pumping of lymph. However, the finding in anaesthetised rats that the treatment was only partially effective compared to a pressure cuff mandates further investigation, as it could be caused by imperfect inhibition of lymphatics and/or by direct venom entry into the vasculature. The greater effectiveness of PBI could be through inhibiting both lymphatic and direct vascular venom toxin absorption. PBI is generally thought of as a technique that inhibits lymphatic function. However, the finding that PBI is effective against envenomation by the *Naja naja*^22^, a cobra with small venom toxins that directly enter the vasculature^23^ and that it inhibits movement of small molecules such as NaI from the site of limb injection^24^ indicates that it also inhibits direct vascular uptake.

The pathways for venom toxin absorption into the circulation subsequent to snakebite have never been unambiguously elucidated for Australian terrestrial elapid venoms, which generally have minimal cytotoxic actions. The consensus view is that they enter the circulation by entry through the lymphatics. This was first inferred from the studies by Barnes & Trueta^23^ on venom from the Tiger snake, an Australian elapid. The authors considered Tiger snake venom toxin components to be > 20 kDa but smaller toxins in this venom have been reported including neurotoxins of about 7 kDa^25^. In contrast, a study on sheep involving subcutaneous injection of an American elapid, *Micrurus fulvius* (Coral snake), which also has minimal cytotoxic actions, presented evidence of both lymphatic and direct vascular entry but whether toxin components accessed both pathways was not determined^26^. The mechanisms underlying direct entry of cobra toxin (*Naja naja*) into the circulation also needs to be elucidated given this venom has tissue damaging components that act at the wound site^23^ and hence toxins may directly enter the vasculature because of cytotoxic disruption of the endothelium.

The pathways for systemic envenomation from snakes in the family Viperidae are likely to utilise both pathways. However, the functionality of these may be rapidly impaired. For example, tissue damaging enzymes such as myotoxin phospholipases (PLA_2_s) have been shown to rapidly inhibit lymphatic function^27^. Direct vascular entry is likely enhanced by snake venom metalloproteinases (SVMPs) which cause inflammation, oedema, haemorrhage, hypovolemia, hypotension and necrosis^27–29^. The mechanisms for direct vascular entry have yet to be fully resolved. The haemorrhagic action, reported to be mediated by P-II and P-III SVMPS targeting capillaries (see^30^) is unlikely to underlie a general increase in vascular permeability given that this is proposed to occur through limited proteolysis of the basement membrane and the action of haemodynamic forces^29^. This would act on pre-capillaries/capillaries under relatively higher pressure but would have minimal effect on capillaries on the venular side or venules where venom uptake occurs. However, there is now evidence that P-III SVMPS directly open endothelial junctions^31^, which could allow a ready pathway for vascular entry of toxin molecules. In contrast, there is at least one venom component present in Indian saw-scaled viper venom (*Echis carinatus*) that can lead to impairment of direct toxin entry into the vasculature through causing neutrophil extracellular trap formation^32^.

We investigated the relative roles of the lymphatic and vascular venom entry pathways consequent to snakebite using venom from the Australian elapid *Pseudonaja textilis*. Our studies depended on establishing a reliable means of impeding lymphatic transport. As cooling from ~35 °C to 24 °C has been shown to approximately halve lymph flow of rat hind limb dermal lymphatics^33^, we investigated the use of more substantial cooling of the test hind limb of anaesthetised rats and found this to markedly inhibit the transport of lymph. This allowed investigating absorption of venom by pathways other than the lymphatics with the finding that direct entry of PTx venom into the vasculature also plays a substantial role. Additionally, we found that PTx venom had a direct effect on vascular permeability as measured by plasma albumin extravasation.

## Results

### Lymph flow is markedly inhibited by cooling

**Figure 1:**
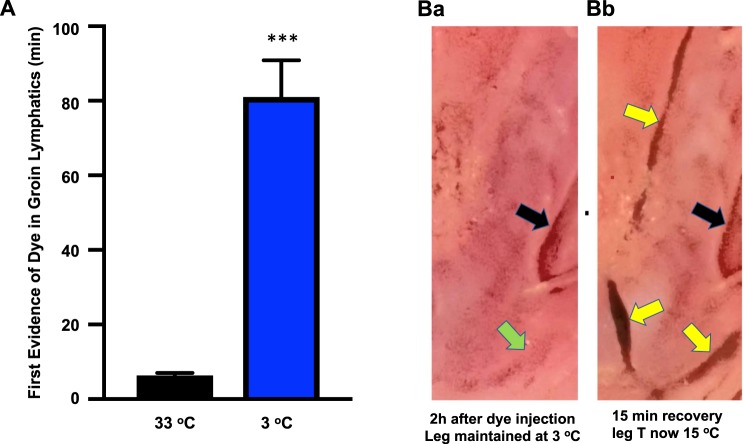
Lymph transit is markedly impeded by cooling. (**A**) Lymph transit time was measured by injecting the marker dye India ink subcutaneously into the hind paw of anaesthetised rats and visually monitoring the time taken to reach the exposed lymphatics in the groin, as indicated by the first evidence of improved visualisation of the vessel wall through greying of the lymph. Data shown as mean ± SEM (P < 0.0001 by unpaired two-tailed t-test or P < 0.001 by non-parametric Mann Whitney test, n = 9 and 7 at mean hind limb temperatures of 33 and 3 °C respectively). (**B**) Contrast-enhanced camera images taken through a dissecting microscope of a rat groin where blood and lymphatic vessels were surgically exposed. (**B**a) Image taken 2 h after injection of India ink into the paw of a rat hind limb where the limb and paw but not the groin were maintained at 3 ± 1 °C. Dye transport was so low that the relatively clear lymphatics were not discerned in the camera image despite being identifiable by eye with the exception of one lymphatic in which there was slight greying (green arrow). (**B**b) Image of the same area taken 15 min after hind limb cooling was removed shortly after the image of Ba. The leg temperature was now at 15 °C. Yellow arrows point to lymphatic vessels that are now discernible because of transport of the India Ink dye. The same filter for contrast enhancement was applied to both images. Black arrows in Ba and Bb point to a shadow at a bifurcation region of a blood vessel. The width of the images is 3 mm.

Experiments were undertaken using cooling as a means of inhibiting the transport of lymph in the test hind limb of anaesthetised rats, as assessed using the marker dye India ink injected into the rat hind paw and measuring the time to reach the exposed groin lymphatics. Cooling to 2.8 ± 0.4 °C caused a13-fold slowing of lymphatic transit time compared to the control (hind limb temperature 33 ± 1 °C; Fig. 1A). These figures were based on the first visual evidence of change in the appearance of the lymphatic wall through greying of the lymph as observed through a dissecting microscope set at optimal magnification to view the surgically exposed groin lymphatics. Unlike control conditions where the density of the ink in the lymphatics rapidly increased to make the lymphatics black (see^20^), the level of darkening was markedly reduced at low hind limb temperatures (i.e. 3 °C) with groin lymphatics only exhibiting the slightest increase in opacity up to 2 h observation. An example is presented in Fig. 1B for an experiment where the hind limb was maintained at 3 ± 1 °C. Arrival of hind paw injected India ink dye in the groin lymphatics was in this case detected 35 min after injection, a time which was the earliest detected (mean time 81 min). This was noted by slightly improved observer discernment of the lymphatic vessel walls through the dissecting microscope. Despite being directly observed through the microscope, the change was so minor that discernment of the groin lymphatic vessels from the other tissue structures was not readily made by standard light photography through the microscope even 2 h after hind paw dye injection with only one lymphatic vessel showing very slight greying (Fig. 1Ba, green arrow). In contrast, lymphatic vessels became discernible by such photography after removal of hind limb cooling with clear visualisation of now black lymphatics, as shown for an image taken 15 min of leg rewarming at which time the leg had rewarmed to 15 °C (Fig. 1Bb, yellow arrows). This indicates that the lymphatics transported negligible volume of dye at low temperatures of about 3 °C.

### Blood plasma venom concentration while slowed in onset is maintained despite cooling

**Figure 2:**
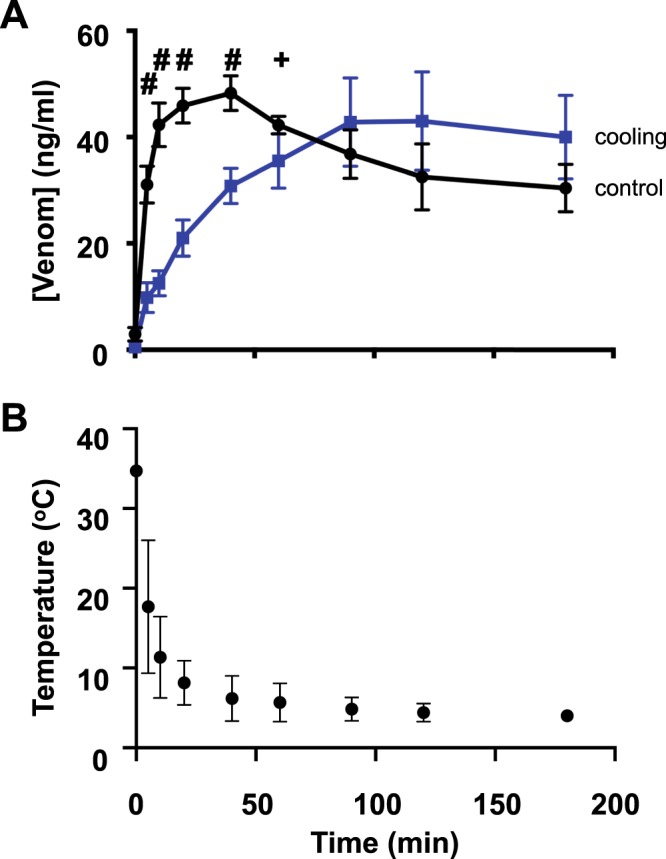
Hind limb cooling slows the onset but does not reduce area under the curve of venom concentrations. (**A**) Mean serum venom concentration measured before and at set times after subcutaneous injection of PTx venom at 0.15 mg/kg into a hind paw of anaesthetised rats without (mean leg temperature 34 ± 1 °C) or with application of hind limb cooling (see Methods) to a mean steady state leg temperature of 4 °C (range 2–6 °C). (**B**) Mean time-course of leg cooling. Serum venom concentrations were determined from 0.2 ml blood samples taken at the times shown. Data shown as mean ± SEM (^#^P < 0.0001, +P < 0.001 two-way ANOVA and unpaired multiple two-tailed t-test; n = 6 for all data points).

The effect that rat hind limb cooling had on serum venom concentration consequent to hind paw injection of PTx venom was examined. We found that the venom concentration consequent to injecting a low dose of PTx venom (0.15 mg/kg) when the test limb was rapidly cooled to a steady state level of 4 °C (Fig. 2B) achieved a similar steady state PTx venom concentration as for control (i.e. test leg at 34 °C) albeit with a slower onset (Fig. 2A). Thus, while cooling slowed the increase in serum venom concentration it had no significant effect on the plateau venom concentration (Fig. 2A) or the area under each curve (mean ± SD: 6670 ± 670 and 6380 ± 1080 (ng/ml).min for control and test respectively). This outcome stands in stark contrast to the India ink experiments, which indicated that there was negligible lymphatic transport of dye at such low hind limb temperatures. Together, these data indicate that there is marked lymphatic-independent (i.e. direct) entry of venom into the vasculature, albeit with slower onset kinetics than when combined with functional lymphatic transport.

### Nitroglycerine ointment slows venom uptake to a similar extent as cooling

**Figure 3:**
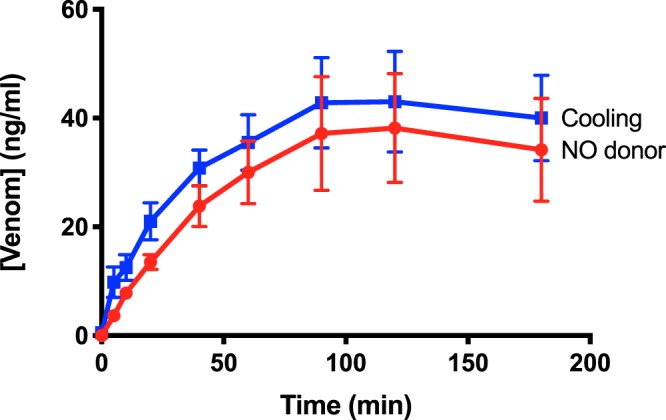
Comparison of the effects of a NO donor and cooling on serum venom concentration consequent to subcutaneous injection of PTx venom at 0.15 mg/kg into a hind paw of anaesthetised rats. Inhibition of the superficial rat hind limb lymphatics with NO donor had a similar effect on serum venom concentration as did complete blockade of hind limb lymphatic function by cooling to near 4 °C (cooling record same as for Fig. 2; data shown as mean ± SEM; not significant difference at any of the time points, two-way ANOVA and unpaired multiple two-tailed t-test).

We have previously reported that application of a nitroglycerine-containing ointment (0.2% wt/wt, Rectogesic, Care Pharmaceuticals, Australia), a treatment that inhibits the superficial lymphatics, slows uptake of PTx venom^20^. We have repeated this experiment but now measuring PTx venom concentration in blood serum. The nitric oxide (NO) donor ointment was applied over the test limb within 1 minute after hind paw injection of PTx venom at 0.15 mg/kg and then reapplied every 15 min for the duration of the experiment with blood samples taken as for the hind limb cooling experiment (Methods). The venom concentration time profile was not significantly different to that for cooling (Fig. 3).

### Toxin entry is maintained despite cooling

**Figure 4:**
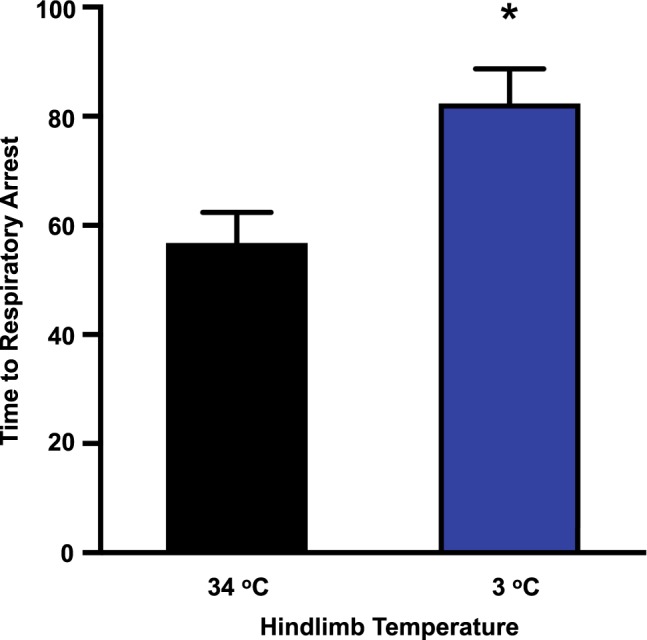
The effect of test hind limb cooling on time to respiratory arrest. Measurements were made consequent to subcutaneous hind paw injection of PTx venom at 1 mg/ml into anaesthetised rats with the test hind limb under control conditions or cooled (mean temperatures of 34 and 3 °C respectively). Data shown as mean ± SEM (*P < 0.05 both by unpaired two-tailed t-test or by non-parametric Mann Whitney test; n = 5 control, n = 5 at 3 °C).

Evidence that venom may directly enter the circulation does not necessarily include toxin molecules given that a polyvalent antibody was used in the EIA assay. A means for assessing this was to investigate the time to respiratory arrest consequent to injection of a lethal dose of PTx venom into the hind paw of anaesthetised rats when lymphatic function was inhibited. To do this venom was injected at 1 mg/kg, a dose known to be lethal to rats under control conditions (i.e. at test hind limb temperature of ~33 °C). Inhibition of hind limb lymph transport by cooling increased the time to respiratory arrest, with a mean increase of 145% for cooling to 3 °C (Fig. 4). This slowing is consistent with inhibition of lymphatic involvement as observed for topical agents that inhibit lymphatics near the skin^20,21^ but the rather small increment in time to respiratory arrest with cooling is inconsistent with the large effect cooling had on inhibiting lymphatic function should the lymphatic system be the only pathway for toxin entry (Fig. 1). In contrast, as we have reported previously, blockade of both pathways for venom entry by application of a pressure cuff completely protected rats against envenomation over the 3 h period monitored^20^. Together these data indicate that both lymphatic and direct vascular pathways underlie entry of the elapid venom toxin molecules.

### Sub-diastolic pressure cuff markedly inhibits both pathways of venom entry

**Figure 5:**
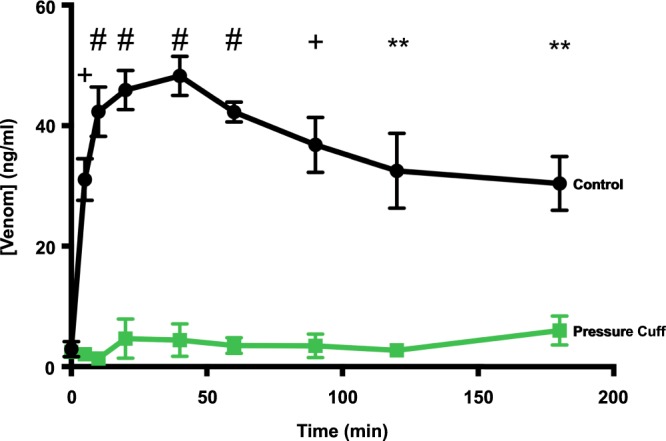
The effect of applying a pressure cuff on the time-course of envenomation. Mean serum venom concentration measured before and at set times after subcutaneous hind paw injection of PTx venom at 0.15 mg/ml without (control) and with a 50 mm Hg pressure cuff. Serum venom concentrations were determined from 0.2 ml blood samples taken at the times shown. Data shown as mean ± SEM (^#^P < 0.0001, +P < 0.001; **P < 0.01, two-way ANOVA and unpaired multiple two-tailed t-test; n = 6 control, n = 4 cuff all points).

Another protocol involved application of a pressure cuff at 50 mm Hg, a pressure below normal rat diastolic pressures of about 80 mm Hg but above typical capillary/venular pressures of < 40 mm Hg^34,35^. The pressure cuff was applied over the test rat hind limb within 20 s after hind paw injection of a low dose of PTx venom (0.15 mg/kg). The serum venom concentration remained very low over the 2 h duration of the experiment indicating that the pressure cuff markedly inhibited venom entry through both pathways (Fig. 5).

### PTx venom causes extravasation of albumin

**Figure 6:**
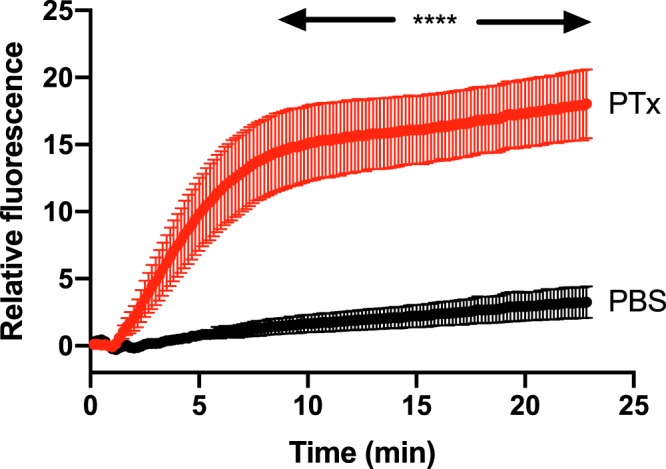
PTx venom increases vascular permeability to plasma albumin. Extravasation of albumin was monitored by pre-injecting anaesthetised Wistar rats with Evans Blue, a dye that binds with high affinity to albumin^47^. Subcutaneous injection of 0.01 mg/kg PTx venom into the depilated lower back skin of these rats caused marked extravasation of the dye measured by its infrared fluorescence response to stimulation with blue light (Methods). Physiological buffered saline (PBS) did not cause a significant change (P < 0.0001 for all data points over the marked region; paired t test; n = 4).

Experiments were made to determine if PTx venom caused extravasation of albumin. This was achieved by determining if the dye Evans blue pre-injected intravenously (IV) into anaesthetised Wistar rats appeared in the interstitium consequent to subcutaneous injection of a non-lethal dose of PTx venom (0.01 mg/kg) through the depilated lower back skin of the albino rats. Figure 6 provides evidence that PTx venom induced extravasation of Evans Blue and hence caused a marked increase in vascular permeability given that albumin has a molecular weight of about 60 kDa.

## Discussion

A start point to this study was establishing a reliable means of inhibiting the transport of lymph in the test rat hind limb that was different to using a pressure bandage with immobilisation. We found that this could be achieved by hind limb cooling to low temperatures and found a marked slowing of lymph transit time (e.g. 13-fold slowing at ~3 °C). This combined with the limited appearance of marker dye (Fig. 1) indicated negligible lymph transport of the dye under these conditions.

In comparison, hind limb cooling of anaesthetised non-recovery rats to a plateau level near 4 °C only slowed the initial rise in the PTx venom concentration time profile and did not significantly alter the area under the venom concentration curve (i.e. total venom load reaching the systemic circulation). Specifically, we found that the venom concentration time profile was slowed taking ~1 h to reach peak concentration whereas under control conditions (i.e. test hind limb at ~35 °C) the peak concentration was achieved with a more rapid onset achieving this within ~10 min. (Fig. 2A). These data support the long-held view that the lymphatics are an important pathway for conducting Australian elapid snake venom to the circulation^12,13,23^ and that inhibiting lymphatic function by cooling provides similar snakebite first aid protection as topical agents that inhibit the superficial lymphatics^20,21^. Significantly, the finding that a plateau component persisted over periods when there was negligible lymphatic flow (i.e. at ~4 °C) indicates that venom is also absorbed directly into the microvasculature. While slower in onset, this pathway for absorption is marked, achieving similar area under the curve to that when lymphatics are functional. Intuitively, this might be expected to be of smaller amplitude given there is now only one transport pathway. The reason for this has yet to be determined but could relate to different rates of vascular absorption when the leg is near 4 °C or the effects of reduced body temperature as despite additional body warming, such cooling of the leg also tended to reduce body temperature (Methods). However, the finding that inhibiting the superficial lymphatics with NO donor ointment made at normal leg and body temperature (i.e. ~35 °C) produced a similar venom concentration profile as cooling (Fig. 3) rules this out, as it suggests that this method is equally effective in inhibiting venom transport by the lymphatics, at least to subcutaneous bites as simulated. Another possible explanation relates to the venom having metalloproteases which could worsen the already discontinuous basement membrane in the end (“initial”) lymphatics possibly leading to increased permeability of the endothelium. Should this occur then lymphatic uptake of venom could decrease over time due to leakage with the vascular pathway becoming dominant for venom absorption,

A key issue is whether direct venom entry into the circulation included toxin molecules, as multiple venom components would be detected by the polyvalent antibody used in the EIA detection system. A means to provide insight into the pathway(s) of PTx toxin entry as compared to general venom entry was to examine the time to respiratory arrest. We found the low temperature protocol caused an ~1.5-fold increase in the time to respiratory arrest (Fig. 4), a value similar to that found for application of NO donor or other agents that inhibit lymphatic function^20,21^. However, while there was some effect in protecting against envenomation, the limited level of protection is inconsistent with a sole lymphatic pathway given that at this low temperature there would be negligible intrinsic lymphatic transport. Thus, if the lymphatics were the only pathway for venom toxin entry then such cooling should have greatly extended rat survival commensurate with that of PBI^12,13^.

The difference in relative success in preventing venom entry into the circulation by a 50 mm Hg pressure cuff compared to inhibiting lymphatic delivery is striking, as the pressure cuff applied to anaesthetised rats (i.e. no movement) effectively abolished venom entry. This is consistent with a dual action of the pressure cuff. Firstly, the pressure cuff under immobilised conditions inhibits lymphatic transport, which as noted above has a dominant role in the initial transport of lymph. Secondly, maintained inhibition of venom in the blood indicates co-suppression of direct vascular uptake. This presumably occurs through acting on capillaries and venules, which typically operate at pressures below that of the pressure cuff^34^. The cuff data also confirm the value of PBI^12,13^ (providing it is applied correctly^17^) or “Pressure Pad”^18,19^ first aid.

Our study has not addressed the nature or size of the toxins. This is of course of considerable importance especially when comparing relative absorption directly into the vasculature compared to the lymphatics. As for all snake venoms, PTx venom contains many toxin components^36^. These include very large toxins such as a prothrombin activator (MWt > 200 kDa^37^), the Kunitz-type protease inhibitor textilotoxin a presynaptic neurotoxin (MWt 70.6 kDa^38^) and short and long three-finger postsynaptic neurotoxins (e.g. Pseudonajatoxin, MWt 12.3 kDa^39^). PTx in rats appears to cause death due to a precipitous fall in respiratory rate^20^ whereas in humans the main venom target is blood coagulopathy^40^. These findings indicate the actions of different toxins. The reasons for these differences are unclear and could relate to relative venom dose or species differences. In humans this is likely to be the very large prothrombin activator whereas in rats the decrease in respiratory rate suggests a central action on respiratory control and hence is likely to be caused by a neurotoxin. At this stage our data confirms that the latter toxin can directly access the vascular system which based on known neurotoxins in PTx venom could range from ~10–70 kDa. Notably, should very large toxins such as prothrombin activator be restricted to the lymphatic pathway then first aid such as topical nitroglycerine-containing ointment which specifically targets the lymphatic pathway may be particularly useful in humans.

A seminal finding of our study is that PTx venom itself has a marked impact on vascular permeability such that now large molecules such as albumin (MWt ~60 kDa) can cross the vessel wall (Fig. 6). This opens up the possibility that a larger size range of toxin molecules (e.g. textilin) may directly permeate the vasculature. This of course depends on whether the extravasation that we measured is not due to a change in permeability of the endothelium caused by physical damage as can be caused by haemodynamic forces consequent to SVMPs^29,30^. However, the rapid time-course and the approximate plateauing of the permeability increase is more consistent with physiological mechanisms such as venom-induced activation of endogenous agents that increase vascular permeability. Clearly this is an area that needs further investigation to determine issues such as: the mechanisms underlying the permeability increase; whether this is a common property for different venoms; and whether the permeability increase occurs on the venular side allowing permeation of large molecules into the vasculature. Positive outcomes may allow development of skin permeable pharmacological agents that prevent direct vascular entry of venom toxins. Such agents combined with existing skin permeable compounds that inhibit lymphatic absorption would represent a useful adjunct to PBI or pressure pad snakebite first aid.

To date, PBI, pressure pad or adjunct treatments such as NO donor ointment are of use as a first aid for bites from snakes with venoms that are minimally cytotoxic such as Australian elapids. The ability to also pharmacologically limit direct vascular entry of venom toxins would add to these procedures. While deaths from Australian elapids are minimal, such agents should be of value for elapids such as Kraits which cause many deaths^41^. Whether these first aid procedures are of benefit for snakes with cytotoxic venoms remains debateable given their propensity to cause local tissue necrosis. There are many such snakes including particularly problematical snakes that cause multiple deaths and morbidity worldwide such as saw-scaled vipers (*Echis sp*.; northern Africa), pit vipers (*Bothrops asper*, *B. atrox*; Central and South America), cobras (*Naja sp*.) and Russell’s viper (*Daboia russelii*; Asia) (see^2,41^). The current rational for such bites is generally to avoid using PBI or equivalent first aid. For example, this applies to bites from North American rattlesnakes (e.g. *Crotalus adamanteus*, *Crotalus atr*ox) on the basis that there are few deaths with victims generally receiving antivenom treatment in time to prevent serious systemic damage^42^.

The case for abandoning such first aid approaches in other countries where the time to get to hospital for antivenom treatment may be longer, needs further consideration. There are examples for and against local venom restriction. A study with application of the pressure pad technique for Russell’s viper bites was found to reduce circulatory venom load compared to when removed^19^. These authors note that in the small number of cases where there was local necrosis, this was not different to untreated victims and that the pads were reasonably tolerated as tested for 1 h and in one case for 2 h. Two PBI studies have been made on pigs envenomated with a lethal dose of Crotalus atrox venom. The first with 10 animals in both the test and control groups reported that PBI applied manually without measurement of bandage pressure gave a 27% increase in survival time, reduced leg swelling but caused a substantial increase in tissue compartmental pressure^43^. The second made with 3 animals in both the test and control groups reported that untreated pigs died in about 14 h, whereas pigs subjected to PBI for 24 h followed by antivenom treatment survived^44^. These authors reported that there was widespread necrosis in the untreated group but only local necrosis in the treated group with pigs reasonably recovering after 7 days. From a theoretical perspective, key issues to consider are venom load and rate of removal. The amount of venom injected can vary substantially but can be large based on viper venom milking (e.g. see^45^). This together with local cytotoxic venom actions to limit absorption through mechanisms such as rapidly inhibiting lymphatic function^27^ and blockage of direct venom uptake into the vasculature through mechanisms such as neutrophil extracellular trap formation^32^ point to clearance of venom from the bite site being limited. Indeed it has been noted from monkey experiments that clearance of venom from a pit viper (Eastern Diamondback Rattlesnake, *Crotalus adamanteus*) can be small with some 90% of injected venom failing to reach the circulation, whereas absorption of an Australian elapid venom (Tiger snake, *Notechis scutatus*) is large 90% being absorbed 4 h after injection^13^. Therefore, first aid procedures that minimise venom entry into the circulation when applied to treatment of cytotoxic snakebite may make relatively small difference to the extent of local tissue damage while saving lives. Better understanding of the pathways by which venoms enter the circulation and developing additional means such as pharmacological agents to inhibit these will help towards this aim. However, the much deeper venom injection inflicted by vipers will limit the ability of skin permeable first aid compounds to be effective and other means of applying such agents will probably be necessary.

In conclusion, our studies demonstrate that intrinsic lymphatic function, namely the absorption and transport of lymph under immobilised conditions (i.e. deeply anaesthetised rats), is negligible at low temperatures (i.e. ≤4 °C). This finding has allowed demonstration in a rat model that venom including toxic components from the Australian elapid *Pseudonaja textilis* rapidly enter the circulation by lymphatic absorption accompanied by slower direct entry into the circulation. Importantly, PTx venom leads to development of plasma albumin extravasation, suggesting that the venom *per se* opens up pathways by which larger PTx venom toxin molecules directly enter the circulation.

## Methods

### Ethics statement

Experimental approval was provided by the University of Newcastle Animal Care and Ethics Committee (Approval A-2009-153) confirming the work fulfils the criteria of the Australian Code of Practice for the care and use of animals for scientific purposes (National Health and Medical Research Council of Australia, 2004).

### Experimental protocols

Male and female Wistar rats (weight range 200–550 g) were used in these studies. All experiments were performed with the animals fully anaesthetised (urethane at 1.5–1.75 g/kg i.p.) and maintained near 37 °C with the rats euthanized without recovery. Experiments involved determining lymph transit times, blood venom concentrations, vascular permeability to albumin or the time to respiratory arrest. Surgery involved exposing groin lymphatics to allow measurement of hind paw to groin lymph transit times and/or cannulation of the carotid artery for measurement of blood pressure and collection of blood for plasma venom assays. Experiments involving cooling were made by immersion of the test hind limb in water at a set temperature or using a cuff through which water at desired temperature was circulated.

Lymph transit time was measured as the time taken for India ink dye injected into the rat hind paw at a volume of 50 μl to first reach the exposed groin lymphatics^20^. Measurements were performed by the same person in all cases who observed the groin lymphatics through a dissecting microscope noting the time of first evidence of dye in the lymphatics. The time point was based on improvement in detection of the groin lymphatics due to the initial arrival of the dye causing better definition of the vessel wall through slight greying of the lymph. Transit times were measured with or without cooling of the test hind limb. Cooling to the desired temperature was established before dye injection.

Venom studies were made by hind paw injection of whole Eastern Brown snake venom (*Pseudonaja textilis* reconstituted from lyophilised form obtained from Venom Supplies, Adelaide, SA) into the rat hind paw. Serum venom concentrations were measured before and as a function of time after hind paw venom injection of PTx at 0.15 mg/kg. Serum venom concentrations were measured by enzyme immunoassay (EIA) using a polyclonal antibody mixture to PTx venom^46^. Serum venom concentration profiles were measured for control (i.e. no intervention) and two experimental conditions that impeded lymphatic transport, these being limb cooling or a limb pressure cuff. Limb cooling experiments were made applying rapid hind limb cooling immediately following hind paw venom injection, a procedure that allowed investigation of the effects of cooling on the changes in serum venom concentration, sub-dermal cooling being relatively rapid, and also the value of cooling as a snakebite first aid for elapid snakebite. Limb cooling to lower temperatures (e.g. 3–4 °C) tended to decrease animal body temperature from 33–37 °C to 29–32 °C despite extra body heating. Pressure cuff measurements were made using a miniaturised sphygmomanometer that allowed application of a set pressure over the test hind limb. Pressure was applied at 50 mmHg within 20 s after injection of venom (test hind limb not cooled). Blood collection involved taking 200 μl samples through a carotid cannula, normally at times 1 min before and then 5, 10, 20, 40, 60, 90, 120, and 180 min after venom injection with corresponding saline infusion made to maintain blood volume. Blood samples were immediately stored on ice and serum samples obtained by centrifugation at the end of the 3 h experiment with samples then stored at −80 °C until EIA analysis (typically 1–4 weeks later).

Time to respiratory arrest measurements involved injecting 20–50 μl of 10 mg/ml PTx venom to give a final dose of 1 mg/kg. Test limb cooling to the desired temperature was established before venom injection.

Vascular permeability to albumin was assessed by fluorescently imaging extravasation of Evans blue on the lower back of anaesthetised non-recovery Wistar rats. Rats were anaesthetised and then the lower back and hindquarters were shaved followed by depilation with a hair removal cream (Nair^TM^, Australia). Evans blue was injected IV at 20 mg/kg. The effects on extravasation of PTx venom was then assessed by subcutaneous injection of 50 ml of PTx venom at 0.01 mg/kg in phosphate buffered saline (PBS) and also injecting an adjacent region with 50 ml of PBS. This experiment was repeated on several rats varying the location of the injections. Extravasation was assessed using the azo dye Evans blue which binds to albumin^47^. This dye is fluorescent when excited by either 488 or 540 nm light with maximal emission at 680 nm^48^, our choice being excitation with 488 nm laser light passed through a diffuser.

### Statistical analysis

Statistical analysis, performed using Graphpad Prism (Graphpad Software, USA), utilised paired or unpaired two-tailed t-test and non-parametric Mann Whitney test where ranks are compared. Some data were analysed using two-way ANOVA with post hoc testing using non-paired two-tailed t tests. Except otherwise noted, data are presented as means ± standard error of the mean (SEM) obtained from independent data points with the number of animals denoted by n.

## Data Availability

Data is housed in the Laboratory of the Corresponding Author (DF van Helden) in the School of Biomedical Sciences & Pharmacy, University of Newcastle, Australia and can be made available on request.
